# SREBPs as the potential target for solving the polypharmacy dilemma

**DOI:** 10.3389/fphys.2023.1272540

**Published:** 2024-01-10

**Authors:** Xue Wang, Yanqiu Chen, Heyu Meng, Fanbo Meng

**Affiliations:** Jilin Provincial Precision Medicine Key Laboratory for Cardiovascular Genetic Diagnosis (Jilin Provincial Engineering Laboratory for Endothelial Function and Genetic Diagnosis of Cardiovascular Disease, Jilin Provincial Molecular Biology Research Center for Precision Medicine of Major Cardiovascular Disease, Jilin Provincial Cardiovascular Research Institute), Department of Cardiology, China-Japan Union Hospital of Jilin University, Changchun, China

**Keywords:** SREBPs, polypharmacy, cardiovascular disease, metabolic syndrome, multimorbidity

## Abstract

The phenomenon of polypharmacy is a common occurrence among older people with multiple health conditions due to the rapid increase in population aging and the popularization of clinical guidelines. The prevalence of metabolic syndrome is growing quickly, representing a serious threat to both the public and the worldwide healthcare systems. In addition, it enhances the risk of cardiovascular disease as well as mortality and morbidity. Sterol regulatory element binding proteins (SREBPs) are basic helix-loop-helix leucine zipper transcription factors that transcriptionally modulate genes that regulate lipid biosynthesis and uptake, thereby serving an essential role in biological systems regulation. In this article, we have described the structure of SREBPs and explored their activation and regulation of signals. We also reveal that SREBPs are intricately involved in the modulation of metabolic diseases and thus have tremendous potential as the novel target for single-drug therapy for multiple diseases.

## 1 Introduction

Multimorbidity refers to the presence of ≥2 chronic diseases, and it is highly prevalent among the elderly population (Salive, 2013). With the rapidly growing geriatric population, over 70% of adults suffer from cardiovascular disease (CVD) by age 70, and over two-thirds suffer from non-CVD complications (Forman et al., 2018). Two important factors that contribute to the CVD pandemic are overweight and obesity, which also give rise to metabolic syndrome (MS) (Sherling et al., 2017). Unfortunately, MS has a very high incidence, and its rising incidence is exacerbating the current polypharmacy problem.

Modern medicine prioritizes the implementation of clinical guidelines. However, it predominantly disregards the trade-offs associated with the long-term balance between advantages and disadvantages, the quality of life in terms of health, the preferences of the patient, and the attainment of goals (Boyd et al., 2005). With the popularization of clinical guidelines, the phenomenon of polypharmacy is more prominent among older adults, who typically exhibit multimorbidity (Masnoon et al., 2017). Approximately 20% of older people in the community who are over the age of 65 consume 10 or more drugs (Hajjar et al., 2005). Elderly patients are at a higher risk of experiencing negative effects from drugs due to changes in how their bodies process and respond to drugs (Carroll and Hassanin, 2017). According to a study, patients who were prescribed ≥8 drugs were 4 times more likely to experience negative effects from the drug. Compared to those who were taking less than 5 drugs (Onder et al., 2010). In the context of this scenario, it is imperative to devise innovative approaches to address multiple risk variables using a single drug. Sterol regulatory element binding proteins (SREBPs) are transcription factors (TFs) that modulate the transcription of lipid synthesis-related genes and are involved in key nodes of signaling pathways affecting numerous physiological and pathophysiological systems (Eberlé et al., 2004). These associations make SREBPs an excellent target for addressing the polypharmacy dilemma.

## 2 SREBPs structure, activation, and regulation

### 2.1 SREBPs structure

**FIGURE 1:**
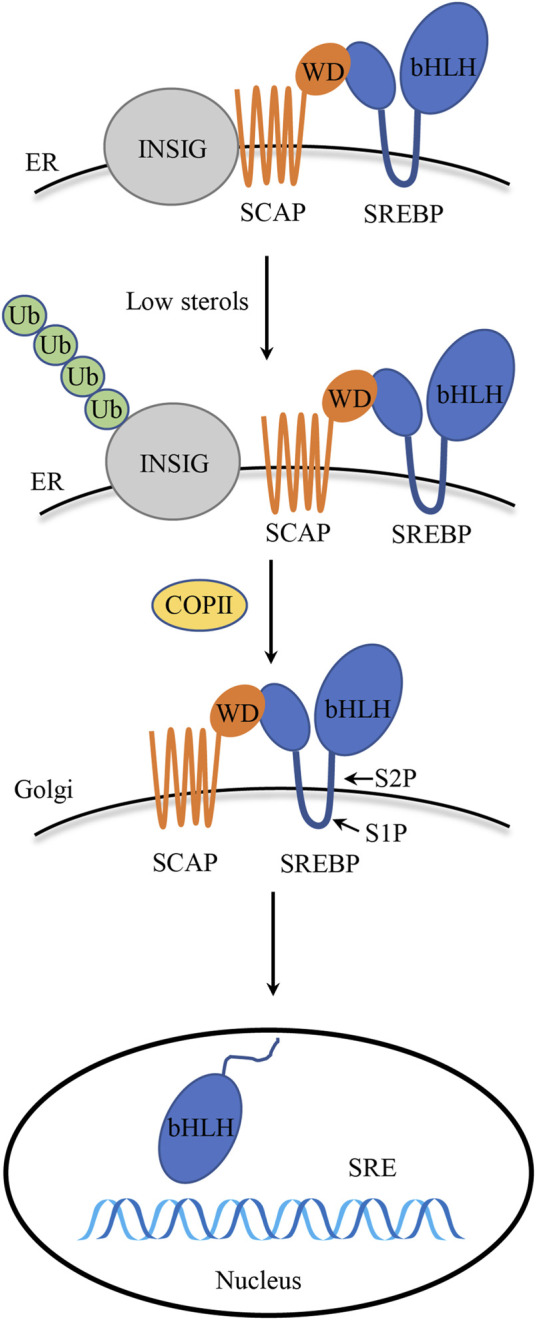
SREBPs structure and activation. Precursor SREBPs bind to ER membrane-bound SCAP. INSIG binds to SCAP to maintain the SCAP/SREBP complex within the ER membrane, particularly in the presence of elevated sterol levels. Once the sterol concentration drops, SCAP detaches from INSIG and escorts SREBPs to the Golgi apparatus. Inside the Golgi apparatus, SREBPs undergo cleavage by S1P and S2P proteases, and the mature N-terminal fragment is released, which, in turn, translocates to the nucleus to initiate transcription of target genes.

SREBPs are TFs that contain an N-terminal TF domain that connects to a C-terminal regulatory domain via a transmembranal hairpin so that both domains face the cytoplasm (Rawson, 2003). They are also in the subcategory of the basic helix-loop-helix leucine zipper (bHLH-Zip). Each nascent SREBP protein has a molecular weight of about 125 kDa and consists of about 1150 amino acids. The N-terminal portion of the SREBPs molecule through the bHLH-Zip region protrudes into the cytosol. The following central portion of SREBPs, the membrane anchoring region, is about 90 amino acids in length. It consists of two hydrophobic, membrane-spanning segments separated by a hydrophilic loop that extends into the lumen of the endoplasmic reticulum (ER) (Weber et al., 2004). The C-terminal regulatory domain contains approximately 590 amino acids responsible for SREBPs subcellular localization and translocation (Xue et al., 2020). Among the principal participants involved in the SREBP pathways, two membrane-based proteins, SREBP cleavage activating protein (SCAP) and insulin-induced gene protein (INSIG), together monitor sterol levels in the ER membrane (Yan et al., 2021). SCAP interacts with INSIG in the ER, and the resulting INSIG/SCAP/SREBP complex resides in the ER (Cheng et al., 2018) (Figure 1). SREBPs come in three forms, namely, SREBP-1a, -1c, and 2, encoded by two genes, SREBF-1 and SREBF-2 (Soyal et al., 2015). Among these, SREBP-1a and 1c are transcribed from the single gene SREBF-1 using distinct promoters and exons (Moon, 2017). The active forms of SREBP-1a and SREBP-1c differ at their extreme N-terminal portion; SREBP-1c lacks 28 amino acids present in SREBP-1a and instead contains 4 unique amino acids of its own (Toth et al., 2004). SREBPs serve many purposes in terms of functionality. For instance, SREBP-1a controls the synthesis and development of lipids, SREBP-1c modifies the synthesis of fatty acids (FASN, ACC, and SCD1) and energy storage, and SREBP-2 regulates the synthesis of cholesterol (HMGCR, DHCR7, and SQLE) and its levels (Shimano and Sato, 2017).

### 2.2 SREBPs activation

Through a feedback system, SREBP activation is closely controlled in order to ensure a suitable response to fluctuations in cholesterol levels. The bHLH-Zip region, which its N-terminal domain comprises, is released proteolytically from the Golgi membrane before it enters the nucleus and initiates transcription (Engelking et al., 2018). In the presence of sterols, the SREBPs precursor and SCAP with eight transmembrane domains form a heterodimeric complex, and the C-terminal region of SCAP containing the WD40 repeat domain extends into the cytoplasm to interact with SREBPs (Gong et al., 2015). Upon sterol deprivation, the SCAP/SREBP complex disassociates from INSIG, carried by COPII and transferred to the Golgi apparatus, and subsequently, INSIG undergoes ubiquitination on lysines 156 and 158, leading to its degradation in proteasomes (Gong et al., 2006). In Golgi apparatus, SREBPs are processed by two consecutive proteolytic cleavages. The S1P protease is responsible for the first cleavage, which takes place inside the short lumen loop. The S2P protease catalyzes a second, fast cleavage of the first transmembrane segment that happens right after the first. As a result, the N-terminal transactivation domain is released, activating genes involved in lipid synthesis and absorption by binding the target gene’s sterol regulatory element (SRE) (Osborne and Espenshade, 2009; Engelking et al., 2018; Su et al., 2019) (Figure 1). The amino acid sequence MELADL in the cytoplasmic loop 6 of SCAP is essential for the interaction of COPII-encapsulated vesicles (Sun et al., 2007). Once cholesterol content exceeds a threshold of 4%–5% of the total lipids in the ER, SCAP interacts with cholesterol, which facilitates its interaction with the ER-resident protein INSIG (Goldstein et al., 2006), which, in turn, keeps COPII from identifying the MELADL sequence in the SCAP loop 6, which ultimately blocks the translocation of the SCAP/SREBP complex to the Golgi apparatus, thus effectively reducing the production of cholesterol and fatty acid (Xiao and Song, 2013).

### 2.3 SREBPs regulation

The INSIG/SCAP/SREBP complexes retention and transport of SREBPs from the ER to the Golgi apparatus are regulated by a variety of signals. Heat shock protein (HSP) 90 is a highly conserved chaperone protein found in numerous tissues. It interacts with over 200 protein substrates called “customers” and regulates their stability, folding, and maturation (Taipale et al., 2010). HSP90 is an emerging SREBP modulator that is associated with the SCAP/SREBP complex, according to recent studies. *In vitro* and *in vivo*, it binds to and stabilizes the SCAP/SREBP complex and controls SREBP activity to preserve lipid homeostasis (Kuan et al., 2017). Effective anticoagulant dipyridamole may significantly increase statin dependence and suppress the growth of tumor cells in models of xenotransplantation and culture (Pandyra et al., 2014). Dipyridamole retains the SREBP precursor in the ER, which effectively overrides normal cellular responses to reduce sterol concentrations and insulin signaling *in vivo*. This, in turn, elevates the ER-localized INSIG-1 protein levels in cultured cells (Esquejo et al., 2021). Phospholate cytosolic phosphoenolpyruvate carboxykinase 1 (PCK1) is a rate-limiting enzyme involved in hepatic and renal gluconeogenesis (Burgess et al., 2007). In human hepatocellular carcinoma cells, phosphorylated PCK1, in turn, phosphorylates INSIG-1 at Ser207 and INSIG-2 at ser151 via GTP as a phosphate donor in the ER. This phosphorylation diminishes interaction between the sterols and INSIG-1/INSIG-2, thereby disrupting the INSIG/SCAP complex, which, in turn, transfers the SCAP/SREBP complex to the Golgi apparatus, thus activating SREBPs and transcription of downstream lipogenesis-related genes (Xu et al., 2020). Cell death-inducing DFF45-like effector B (Cideb) is mostly found in the hepatic ER and lipid droplets (Ye et al., 2009), and it is known to form the COPII complex by connecting the SREBP/SCAP complex to the ER outlet. This enhances the SREBP/SCAP complex loading into COPII vesicles for their delivery to the Golgi apparatus under conditions of sterol deprivation (Su et al., 2019) (Table 1).

**TABLE 1:** Regulation of the SREBP pathway.

Items	Targets	Functions	Lipid metabolism
HSP90	SCAP/SREBP complex	HSP90 inhibition led to proteasome-dependent degradation of the SCAP/SREBP complex, which resulted in the downregulation of SREBPs target genes (Kuan et al., 2017)	**↑**
Dipyridamole	INSIG/SCAP/SREBP	Dipyridamole increases the stability of ER-localized INSIG and blocks SCAP/SREBP complex ER-to-Golgi trafficking (Esquejo et al., 2021)	**↓**
PCK1	INSIG	Phosphorylated PCK1 translocates to the ER, where it phosphorylates INSIG to reduce the binding with sterols and disrupts the interaction between INSIG and SCAP (Xu et al., 2020)	**↑**
Cideb	SCAP/SREBP complex	Cideb selectively promotes the loading of the SREBP/SCAP complex into COPII vesicles (Su et al., 2019)	**↑**

## 3 SREBPs and metabolic diseases

### 3.1 SREBPs and metabolic dysfunction–associated steatotic liver disease (MASLD)

MASLD is now recognized as the most prevalent hepatic disorder, affecting approximately 1.7 billion people worldwide. It involves a series of liver abnormalities ranging from nonalcoholic fatty liver to nonalcoholic steatohepatitis (Friedman et al., 2018). MASLD represents the presence of excess fat accumulation in the liver without consumption of excess alcohol. Most MASLD patients suffer from metabolic comorbidity, for example, insulin resistance (IR), type 2 diabetes mellitus (T2DM) and obesity, which greatly increases their risk of CVD and extrahepatic cancer (Angulo et al., 2007; Chalasani et al., 2012; Zhou et al., 2020). Its major pathological progression follows the “triple strike” process, namely, steatosis, lipotoxicity, and inflammation (Cobbina and Akhlaghi, 2017). The liver serves an important part in the metabolism of lipids. Furthermore, it also plays a crucial role in regulating lipid balance by controlling the production of new fatty acids, their transportation to other tissues, and their use as a source of energy (Nguyen et al., 2008). Triglyceride (TG) overaccumulation in the liver is a hallmark of MASLD, and it is caused by dysregulated hepatic fatty acid metabolism due to alterations in intake, synthesis, secretion, or degradation (Nguyen et al., 2021).

**FIGURE 2:**
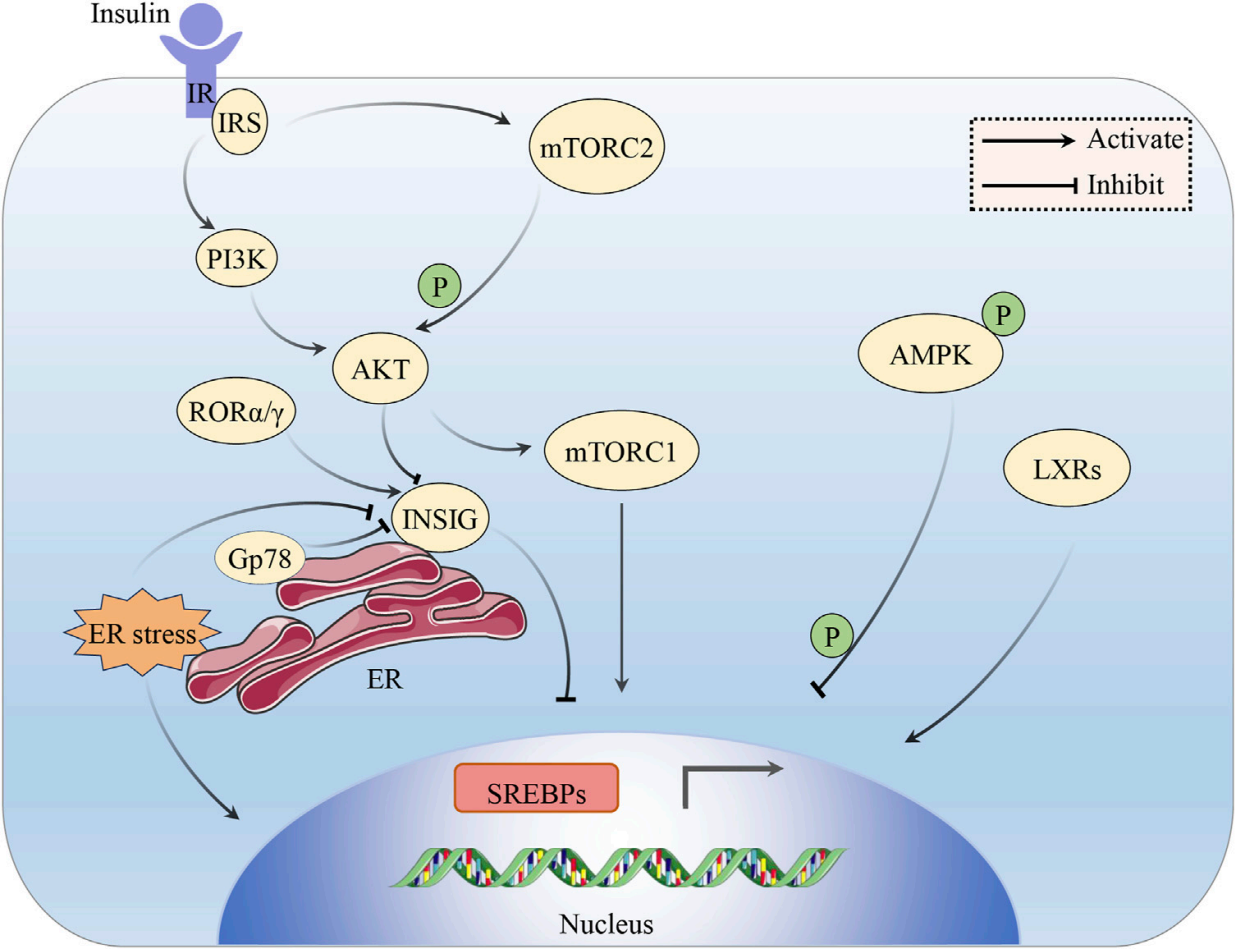
The impact of insulin on the SREBP pathway. Insulin signaling activates SREBPs through the PI3K/AKT/mTORC1 pathway. AKT also inhibits the involvement of INSIG in the activation of SREBPs. AKT can be phosphorylated and activated by mTORC2. LXRs can directly activate the expression of SREBP-1c. Phosphorylated AMPK can impede SREBP-1c cleavage and nuclear translocation. ROR α/γ serves to inhibit SREBP by stimulating INSIG. ER stress can induce the cleavage of SREBP-1c and rapid degradation of INSIG. Gp78 mediates the degradation of INSIG in the liver.

The stimulating effect of insulin on lipogenesis in the liver and adipose tissue is well known, but its cellular mechanisms are not well understood. The mammalian target of rapamycin (mTOR) is a bispecific protein kinase that phosphorylates serine/threonine and tyrosine residues (Yin et al., 2016) to regulate cell growth, survival, metabolism and immunity (Hua et al., 2019). mTOR plays an essential role as a core component of two functionally distinct multi-subunit protein complexes called mTORC1 and mTORC2 (Murugan, 2019). In hepatocytes, mTOR is activated by insulin and nutrients through the inositol phosphate 3-kinase (PI3K)/AKT pathway, and its complex mTORC1 promotes SREBP-1 activity and SREBP-1c overexpression in the liver by inducing SREBP-1 expression, processing, and nuclear accumulation, which, in turn, promotes TG accumulation (Bakan and Laplante, 2012). However, in the absence of Akt signaling, the activation of mTORC1 alone is inadequate to stimulate liver SREBP-1c. Additional research has revealed that liver SREBP-1c and lipogenesis also depend on AKT to inhibit INSIG directly (Yecies et al., 2011). mTORC2-mediated AKT Ser473 phosphorylation regulates hepatic glucose and lipid metabolism to control whole-body metabolic homeostasis (Hagiwara et al., 2012). The liver X receptors (LXRs), LXRα and LXRβ belong to the nuclear hormone receptor superfamily of ligand-activated TFs. Endogenous LXR agonists, such as cholesterol derivatives, including oxidized forms of cholesterol, cholesterol precursors, and plant sterols, directly bind to the LXR ligand binding domain (Schulman, 2017). In the liver, LXRs can directly activate the promoter of SREBP-1c to stimulate hepatic lipogenesis, leading to a significant increase in hepatic TG content (Schultz et al., 2000; Song et al., 2018). AMPK promotes the phosphorylation of Ser372, inhibits the cleavage and movement of SREBP-1c into the nucleus, and suppresses the expression of target genes regulated by SREBP-1c in hepatocytes exposed to high glucose. This results in a decrease in the production of fats and the accumulation of lipids (Li et al., 2011). The expression of SREBP-1c in the liver also displays daily rhythmicity (Saran et al., 2020). For example, the nuclear receptors RORα and RORγ, key components of the molecular circadian clock, control the circadian expression of INSIG-2, which keeps feeding-induced SREBP-1c activation under check (Zhang et al., 2017) (Figure 2) (Table 2).

**TABLE 2:** The roles and molecular mechanisms of the SREBP pathway in different metabolic diseases.

Disease type	Targets	Mechanisms
MASLD	PI3K/AKT/mTORC1/SREBP-1c	Insulin and nutrients activate the pathway to promote TG accumulation (Bakan and Laplante, 2012)
	AKT/INSIG	mTORC1 cannot stimulate lipogenesis alone and Akt involves the suppression of a liver-specific isoform of INSIG is also required (Yecies et al., 2011)
	mTORC2/AKT/SREBP-1c	Hepatic mTORC2 inhibition results in decreased glucose metabolism caused by decreased Akt activity and subsequently reduced SREBP-1c and glucokinase activities (Hagiwara et al., 2012)
	LXRs/SREBP-1c	LXRs directly activate the promoter of SREBP-1c to stimulate hepatic lipogenesis, leading to a significant increase in hepatic TG content (Song et al., 2018)
	AMPK/SREBPs	AMPK binds to and phosphorylates SREBP-1c and SREBP-2 to prevent its autoregulation and transcription of target lipogenic genes (Li et al., 2011)
	ROR/INSIG-2/SREBP-1c	Loss of hepatic RORs led to a marked induction of SREBP-1c protein, which was induced by the reduction of INSIG-2 expression (Zhang et al., 2017)
DM	PI3K/AKT/PKB/SREBP-1c	Under IR conditions, insulin regulates SREBP-1c-dependent lipogenesis *via* the PI3K/AKT/PKB pathway, leading to obesity, MS, and T2DM (Sajan et al., 2018)
	ER stress/INSIG-1/SREBP	During ER stress, degradation of INSIG-1 results in the proteolytic activation of SREBP (Kammoun et al., 2009)
	Gp78/INSIG/SREBP	Knockout of gp78 results in elevated levels of INSIG-1/2, suppression of SREBP, reduction in lipid synthesis, and protection of mice from the effects of diet/age-induced obesity and glucose intolerance (Liu et al., 2012)
	TGFβ1/SREBP-1/Smad3	The activation of SREBP-1 by TGFβ1 in glomerular mesangial cells is an important regulator of Smad3-mediated gene transcription (Chen et al., 2014)
	SREBPs	Overexpression of SREBPs in pancreatic β-cells can affect β-cell function, leading to diabetes-related diseases (Takahashi et al., 2005; Ishikawa et al., 2008; Iwasaki et al., 2009)
Atherosclerosis	FGF21/SREBP-2	FGF21 could inhibit atherosclerosis by inhibiting hepatic SREBP-2 to reduce Hypercholesterolemia (Lin et al., 2015)
	SREBP-2/NLRP3	SREBP-2 activation of NLRP3 inflammasome in endothelium mediates hemodynamic-induced atherosclerosis susceptibility (Xiao et al., 2013)
	AMPK/SREBPs	Inhibition of SREBPs by AMPK in the liver could produce anti-atherogenic changes (Li et al., 2011)
Hyperlipidemia	SREBP-1c	When SREBP-1c is activated in mice’s liver, it can lead to an increase in TG synthesis and result in hyperlipidemia (Okazaki et al., 2010)

Recent findings indicate that increased levels of insulin during IR stimulate the production of fat through the activation of SREBP-1c. This process contributes to the development of MASLD in both human beings and animal models (Nguyen et al., 2021). Transgenic animal investigations have demonstrated that hyperinsulinemia in ob/ob mice triggers hepatic SREBP-1c activation, leading to liver steatosis (Shimomura et al., 1999). In contrast, mice with hepatic SREBP-1c deletion exhibit reduced hepatic TG by approximately 50% (Moon et al., 2012). The *in vivo* role of SREBP-1c was demonstrated in a transgenic mice model that overexpresses SREBP-1c in the liver, which led to the development of hepatic steatosis due to the increase in lipogenesis (Shimano et al., 1997). When SREBP-2 was overexpressed in the liver of transgenic mice, there was a significant increase in cholesterol synthesis (Horton et al., 1998).

### 3.2 SREBPs and diabetes mellitus (DM)

Due to significant changes in contemporary human living, the prevalence of DM is increasing fast worldwide (Ogurtsova et al., 2017). The prevalence of both DM and prodromal DM in the United States exceeds 50% (Menke et al., 2015). Currently, the precise pathophysiology of DM is still somewhat uncertain. However, many mechanisms, including IR, β-cell dysfunction, and apoptosis, with low circulating insulin levels, oxidative stress, mitochondrial dysfunction, and inflammation are associated with DM (Yaribeygi et al., 2019). IR is an important component of MS and a risk factor for diseases such as diabetes, CVD, and Alzheimer’s disease (Onyango, 2018).

In hepatocytes, insulin regulates glucose metabolism via the PI3K/AKT pathway. Once insulin interacts with the cell membrane-based tyrosine kinase insulin receptor, the insulin receptor substrate (IRS) becomes phosphorylated, which, in turn, activates PI3K, which then activates the AKT/protein kinase B (PKB) activity, a kinase that controls numerous anabolic events and further activates downstream pathways (Manning and Cantley, 2007; Sajan et al., 2018; Patel and Goyal, 2019). From mRNA transcription to protein degradation, AKT might influence SREBP at numerous levels. Under conditions of IR, hepatic gluconeogenesis is no longer suppressed, which results in enhanced hepatic glucose output, hyperinsulinemia, and augmented SREBP-1c-dependent lipogenesis, which leads to obesity, MS, and eventually T2DM development (Sajan et al., 2018). ER stress can induce proteolytic SREBP-1c cleavage and rapid degradation of INSIG to activate the whole lipogenic program (Kammoun et al., 2009). Gp78 is a ubiquitin ligase anchored on the ER, mediating the degradation of INSIG in the liver. The disruption of gp78 in the liver enhances the inhibitory effect of INSIG on SREBPs, resulting in a reduction of the SREBP pathways, thus protecting mice from diet/age-induced obesity and glucose intolerance (Liu et al., 2012) (Figure 2).

As a serious complication of DM, diabetic nephropathy (DN) is one of the main causes of end-stage renal failure (Su et al., 2020). Levi et al. found that the expression of SREBP-1 and SREBP-2 increased in renal tissues of db/db mice, and total cholesterol and TG were deposited in the kidney, resulting in glomerulosclerosis, tubulointerstitial fibrosis and proteinuria (Wang et al., 2005). Chen et al. found SREBP-1 as a major regulatory factor in TGFβ1-mediated fibrotic kidney disease, where interaction with Smad3 and CBP may serve as a potential novel therapeutic target for the treatment of DN (Chen et al., 2014) (Table 2). β-Cell lipotoxicity is closely related to the T2DM (Lytrivi et al., 2020). The study discovered that when SREBPs were overexpressed in pancreatic β-cells of transgenic mice, it resulted in a decrease in the number and size of islets, as well as a drop in insulin content. This led to reduced insulin secretion and a little decline in glucose tolerance (Iwasaki et al., 2009). The activation of SREBP-1c causes β-cell dysfunction, leading to impaired glucose tolerance or mild diabetes (Takahashi et al., 2005). Activation of SREBP-2 leads to reduced β-cell mass and impaired insulin secretion due to cholesterol accumulation, resulting in severe diabetes (Ishikawa et al., 2008).

Based on the data mentioned above, the development of drugs that concentrate on SREBP offers immense promise for diabetes patients with IR.

### 3.3 SREBPs and atherosclerosis

CVD is a prevalent health issue in contemporary society. Atherosclerosis is the primary etiology of CVD and has been associated with a high rate of mortality (Torres et al., 2015). Atherosclerosis refers to the accumulation of fat and/or fibrotic substances in the intima of arteries. It is characterized by the formation of lipid and (immune) cellular plaques in the intima of large and medium-sized arteries (Libby et al., 2013). Atherosclerotic CVD was previously considered to be present mostly in industrialized countries, but now it has spread all over the world (Libby, 2021). The resulting ischemic heart disease is a major contributor to the global disease burden, with devastating consequences for human life and health (Virani et al., 2020).

In the early stage of atherosclerotic lesions, low-density lipoprotein (LDL) accumulation in the intima is oxidized or otherwise modified (Ference et al., 2017). Monocytes then accumulate within the intima then differentiate into macrophages, which engulf the modified lipoproteins to form foam cells (markers of early fatty streak lesions) (Tabas et al., 2015), which, in turn, induce inflammation (Libby et al., 2019). Activated endothelial cell-mediated release of numerous chemokines and growth factors, as well as the macrophage-mediated induction of extracellular matrix component proliferation and synthesis within the intimal chamber, results in muscle fiber plaque formation (Gimbrone and García-Cardeña, 2016).

Recent research shows that SREBPs may have a role in the development of atherosclerosis through various pathways. Fibroblast growth factor 21 (FGF21) is a peptide hormone produced by different organs, and it modulates energy homeostasis (Fisher and Maratos-Flier, 2016). Lin et al. found that FGF21 could prevent atherosclerosis by suppressing hepatic SREBP-2 and induction of adiponectin in mice (Lin et al., 2015). Atherosclerosis can preferentially develop within the branches and bends of the arterial tree. At the cellular and molecular level, the turbulence mode with low shear stress markedly increases inflammation- and oxidative stress-related gene expression (Chiu and Chien, 2011). Xiao et al. demonstrated that the atheroprone flow induces NLRP3 inflammatory body in the endothelium via SREBP-2 activation. This, in turn, increases the endothelial innate immunity, which works in synergy with hyperlipidemia, to promote susceptibility to atherosclerosis (Xiao et al., 2013). In animal experimentations, multiple drugs were found to improve mice atherosclerosis by inhibiting the SREBP-related pathways. For instance, synthetic polyphenol s17834 activates AMPK, binds to SREBP-1c and SREBP-2, and phosphorylates SREBP-1c and SREBP-2 to attenuate mice atherosclerosis (Li et al., 2011). Likewise, the small molecule betulin induces an association between the SCAP and INSIG to effectively inhibit SREBP maturation, thereby reducing atherosclerotic plaque size while improving plaque stability (Tang et al., 2011). Several case-controlled studies reported more interesting experimental results. Li and others reported that the SREBP-1 and SREBP-2 expressions were upregulated in the peripheral plasma of patients with coronary artery disease (Li et al., 2020). On the other hand, Peng et al. discovered that the levels of SREBP-1 transcripts in the peripheral blood leukocytes of patients with coronary artery disease were significantly lower compared to individuals without coronary artery disease. Furthermore, patients with high-risk, complicated coronary artery disease had a significant decrease in SREBP-1 levels (Peng et al., 2019) (Table 2).

Several studies have confirmed the intricate and direct correlation between SREBPs and atherosclerosis. Furthermore, the regulation of atherosclerosis may involve processes that are different from those that regulate serum lipids.

### 3.4 SREBPs and hyperlipidemia

Hyperlipidemia refers to the unusually high concentrations of serum lipids or lipoproteins owing to dysregulated fat metabolism or activity. This is typically brought on by eating disorders, obesity, genetic diseases (ex. familial hypercholesterolemia), or other diseases like diabetes (Sudhakaran et al., 2018), as well as dysregulated LDL and high density lipoprotein (HDL), hypertriglyceridemia and mixed hyperlipidemia (Karr, 2017). Hyperlipidemia is a common metabolic disease that contributes greatly to CVD, T2DM, atherosclerosis, hypertension, and MASLD occurrences (An et al., 2020).

SREBPs are core players that modulate lipid biosynthesis. Currently, many studies have found that regulation of the SREBP pathways can affect hyperlipidemia. Li et al. discovered that in high-fat, high-sucrose diet-fed obese LDLR^−/−^ mice, there was a significant increase in both SREBP-1 and SREBP-2 in the liver (Li et al., 2011). Similarly, the activation of SREBP-1c in the liver of mice resulted in an elevated production of TG, which could potentially cause hyperlipidemia (Okazaki et al., 2010). Currently, there has been extensive study on the involvement of SREBP-related pathways in the regulation of hyperlipidemia. Developing new drugs that target these pathways could be a promising strategy for treating hyperlipidemia (Table 2).

## 4 SREBPs and inhibitors

Inhibition of the SREBP pathways will lower the risk of metabolic diseases. Currently, research on inhibitors targeting SREBPs and their related pathways is ongoing, including various extracts, small molecule compounds, etc. The complicated regulation of SREBPs indicates that different strategies can be developed. These methods include stimulating the interaction between SCAP and INSIG, increasing INSIGs, depleting SCAP, inhibiting the S1P or S2P, micro-RNAs (miRNAs), and accelerating the degradation of nuclear (n)-SREBP (Tang et al., 2011). The discovery and research of SREBP inhibitors make it possible to develop drugs targeting related targets, and inhibition of this pathway provides a prospect for the treatment of CVD and its related metabolic diseases.

### 4.1 Betulin

Betulin is a natural pentacyclic lupine-structured triterpenoid. Its extraction in 1788 was the first of its kind from a plant source, and it was shown to have numerous pharmacological properties (Fulda, 2008). Betulinic acid is an essential natural derivative produced by betulin oxidation (Tuli et al., 2021). The betulin and betulinic acid structures possess poor water solubility. At present, there are many reports on the methods of preparing new dosage forms and derivatives of betulin and betulinic acid so as to improve their therapeutic effects on cellular and animal models by enhancing their poor water solubility and targeting the required cell lines (Amiri et al., 2020).

Fortunately, extensive research has been conducted on the effects of betulin and its derivatives on SREBPs. In metabolic diseases, betulin was reported to have excellent potential as a single drug for treating multiple diseases. Studies revealed that suppression of the nuclear form of SREBPs by long-term treatment with betulin enhances the expression of ATP-binding cassette protein A1 (ABCA1) and G1 (ABCG1) in macrophages and promotes cholesterol efflux to suppress atherosclerosis *in vitro* and *in vivo* (Gui et al., 2016). Moreover, Bai and others demonstrated that betulin attenuates alcoholic liver injury by blocking SREBP-1 regulation of fatty acid synthesis and activation of the SIRT1/LKB1/AMPK axis (Bai et al., 2016).

Betulin is a specific inhibitor of the SREBP pathways. Tang et al. discovered that administering betulin to C57BL/6J mice in doses of 15 or 30 mg/kg/day for 6 weeks or 30 mg/kg/day for 14 weeks in LDLR^−/−^ mice that were fed a western-type diet resulted in decreased cholesterol and TG levels and improved insulin sensitivity, ultimately preventing atherosclerosis *in vivo*. Betulin bound to SCAP, which increased the interaction between SCAP and INSIG, thereby inhibiting the maturation of SREBP (Tang et al., 2011) (Table 3). Currently, clinical trials involving betulin and its derivatives primarily center around skin, tumor, or anti-anxiety experiments (Gielecińska et al., 2023). It is desirable that in the future, greater emphasis will be placed on utilizing betulin for the treatment of metabolic diseases.

**TABLE 3:** The mechanism and effects of SREBP inhibitors.

Items	Targets	Mechanism	Dosage	Effect	References
Betulin	INSIG/SCAP	Combines with SCAP to enhance the interaction between SCAP and INSIG, thereby inhibiting the maturation of SREBP.	Western-type diet C57BL/6J mice were treated with betulin 15 or 30 mg/kg/day for 6 weeks	Reduced cholesterol and TG levels and increased insulin sensitivity	Tang et al. (2011)
			Betulin 30 mg/kg/day treated for 14 weeks for LDLR^−/−^ western type die mice	Prevention of atherosclerosis	
Fatostatin	SCAP/SREBP complex	Directly bind to SCAP, retain the SCAP/SREBP complex in ER, and block its transport to the Golgi apparatus	Fatostatin 30 mg/kg/day was intraperitoneally injected into standard laboratory chow ob/ob mice for 28 days	Reduced weight, blood sugar, and liver fat accumulation	Kamisuki et al. (2009)
KK-052	SCAP/SREBP	Reduce the levels of endogenous SREBP and SCAP.	KK-052, 10 mg/kg treated ob/ob mice, 5 times a week for 4 weeks	Reduced liver steatosis, serum TG, and glucose	Kawagoe et al. (2021)
25HC3S	LXR/SREBP-1c	Reduce lipogenesis by inhibiting the LXR/SREBP-1c signaling pathway	C57BL/6J mice fed a high-fat diet were injected twice with 25HC3S (25 mg/kg) intraperitoneally and fasted for 14 h for acute treatment	Significantly reduced serum TG and cholesterol levels	Xu et al. (2013)
			Once every 3 days for 6 weeks and fasted for 5 h for long-term treatments	Reduced lipid levels in liver tissue of MASLD mice	

### 4.2 Fatostatin

Fatostatin is a diarylthiazole compound that was originally identified from a synthetic small molecule library as a chemical inhibitor blocking insulin-induced adipogenesis (Choi et al., 2003). A study demonstrated that fatostatin blocked SREBP cleavage and target gene expression. In addition, fatostatin prevented Golgi apparatus modification of SCAP N-linked glycans, suggesting that fatostatin blocked SREBP activation by inhibiting the ER-to-Golgi transport of SCAP (Shao et al., 2016).

Kamisuki et al. found ob/ob mice fed standard laboratory chow and daily injected with fatostatin at a dose of 30 mg/kg for 28 days demonstrated decreased body weight and blood glucose levels. The inhibition of SREBP by fatostatin downregulated lipogenic enzymes, enhanced fatty acid oxidation, reduced weight, and increased insulin sensitivity, which caused a lower level of glucose (Kamisuki et al., 2009) (Table 3). Fatostatin or its analogs, therefore, may serve as a chemical tool that provides insights into the regulation of the SREBP pathways.

### 4.3 Vitamin D

Vitamin D is critical for the modulation of metabolism, calcium, and phosphorus absorption for bone health. Interestingly, it is also reported to regulate mechanisms other than mineral homeostasis and bone health maintenance (DeLuca, 2004). There are two different forms of vitamin D: vitamins D_2_ and D_3_. Vitamin D (D_2_ and D_3_) acquired from the skin and diet undergo two sequential hydroxylations: first in the liver (25[OH]D) and then in the kidney, leading to its biologically active form 1,25-dihydroxyvitamin D (1,25[OH]_2_D) (Alshahrani and Aljohani, 2013). Based on current reports, vitamin D_3_ metabolites modulate mammalian gene expression using two distinct networks: the vitamin D receptor (VDR) and the SREBP pathway (Nagata et al., 2019).

In fact, the vitamin D_3_ metabolite 25[OH]D_3_ reduces SREBP levels independent of VDR. It is directly associated with SCAP and inhibits the SREBP response gene expression by inducing SCAP protein hydrolysis and ubiquitin-mediated degradation (Asano et al., 2017). However, the therapeutic effectiveness of 25[OH]D_3_ is diminished because of its VDR-mediated calcification impact. In light of this circumstance, scientists have created KK-052, the initial SREBP inhibitor based on vitamin D. KK-052 maintained the ability of 25[OH]D_3_ to induce the degradation of SREBP but lacked in the VDR-mediated activity. Administering 10 mg/kg of KK-052 to ob/ob mice five times weekly for 4 weeks noticeably decreased their body weight and improved liver steatosis (Kawagoe et al., 2021) (Table 3).

Currently, vitamin D_2_ and vitamin D_3_ are widely used in the treatment of different diseases. However, research has shown that higher vitamin D intake was associated with a lower risk of CVD in men (Sun et al., 2011). However, some studies indicate that supplementing high doses of vitamin D every month may not prevent CVD (Scragg et al., 2017). Therefore, new compounds with distinctive chemical structures and pharmacological properties, such as KK-052, offer the potential to fulfill our particular requirements in treating metabolic diseases.

### 4.4 25-Hydroxycholesterol (25-HC) and 25-hydroxycholesterol 3-sulfate (25HC3S)

Cholesterol 25 hydroxylase can convert cholesterol to a secondary side chain oxysterol called 25-HC (Wang et al., 2019a). 25-HC is involved in numerous processes, including inflammation, immune responses, and cancer development (Wang et al., 2019b). In the last few decades, the roles of 25-HC in cholesterol and bile acid metabolism, antiviral process, inflammatory and immune response, and survival pathways have been extensively elucidated (Cao et al., 2020). One study reported that 25-HC is more effective than cholesterol in inhibiting SREBP-2. Although SCAP can sense cholesterol, 25-HC strongly associates with INSIG, which, in turn, enhances the interaction between SCAP and SREBP-2 (Goldstein et al., 2006), therefore minimizing the production of cholesterol. It is important to mention that 25-HC is a well-known agonist of LXRs, and when LXRs are activated, it can trigger the synthesis of ABCA1, ABCG1, and SREBP-1c. This, in turn, leads to a decrease in atherosclerosis and an increase in lipid buildup in the liver (Liu et al., 2018).

A novel sulfated oxysterol, 5-cholesterin-3β, 25-diol 3-sulfate (sulfated 25-hydroxycholesterol, 25HC3S) has been identified in hepatocytes overexpressing the mitochondrial cholesterol delivery protein, StarD1 (Ren et al., 2006). 25HC3S is also known as LXR antagonist. Bai et al. found that 25HC3S significantly reduced serum and hepatic lipid levels by inhibiting the LXR/SREBP-1c signaling pathway (Bai et al., 2012). Xu et al. found that C57BL/6J mice fed a high-fat diet were intraperitoneally injected with 25HC3S (25 mg/kg) twice and fasted overnight (14 h) for acute treatment, significantly reducing serum TG and cholesterol levels or once every 3 days for 6 weeks and fasted 5 h for long-term treatments reduced lipid levels in the liver tissue of the MASLD mouse model (Xu et al., 2013) (Table 3). DUR-928 is an endogenous form of 25HC3S. It has been found to inhibit lipid biosynthesis by suppressing the LXR/SREBP-1c pathway, suppressing inflammation by reducing inflammatory mediators, and enhancing cell survival by inhibiting apoptosis (Hassanein et al., 2023). Currently, DUR-928 is undergoing clinical trials and has shown great potential for the treatment of alcohol-associated hepatitis (Wang et al., 2020).

### 4.5 Other inhibitors

The alkaloid lycorine, isolated from the plant Narcissus in 1877 (Roy et al., 2018), binds to SCAP and inhibits the SREBP pathway to ameliorate high-fat diet-induced hyperlipidemia, hepatic steatosis and IR in mice (Zheng et al., 2021). Zexie Tang (ZXT) is a classical Chinese medicine prescription from Synopsis of the Golden Chamber. Xie et al. found that ZXT has excellent lipid-lowering effects by inhibiting SREBPs (Xie et al., 2022).

## 5 SREBPs and miRNAs

In addition to transcriptional regulation, miRNAs can also modulate lipid metabolism via post-transcriptional regulation (Moore et al., 2010). miRNAs belong to a large family of small non-coding RNAs, and they are expressed in animals, plants, and some viruses. miRNAs negatively regulate mRNA stability (Saliminejad et al., 2019). When miRNAs bind with specific locations in the 3′untranslated region of mRNAs that complement them, they inhibit translation by causing the degradation of the associated mRNA (Bartel, 2009). Functional studies have demonstrated that miRNAs play several crucial roles in physiological processes such as developmental timing, cell differentiation, embryogenesis, metabolism, organogenesis, and apoptosis (Ha and Kim, 2014). Current studies revealed that many miRNAs are involved in the regulatory process related to the SREBP pathways.

### 5.1 miR-33

**FIGURE 3:**
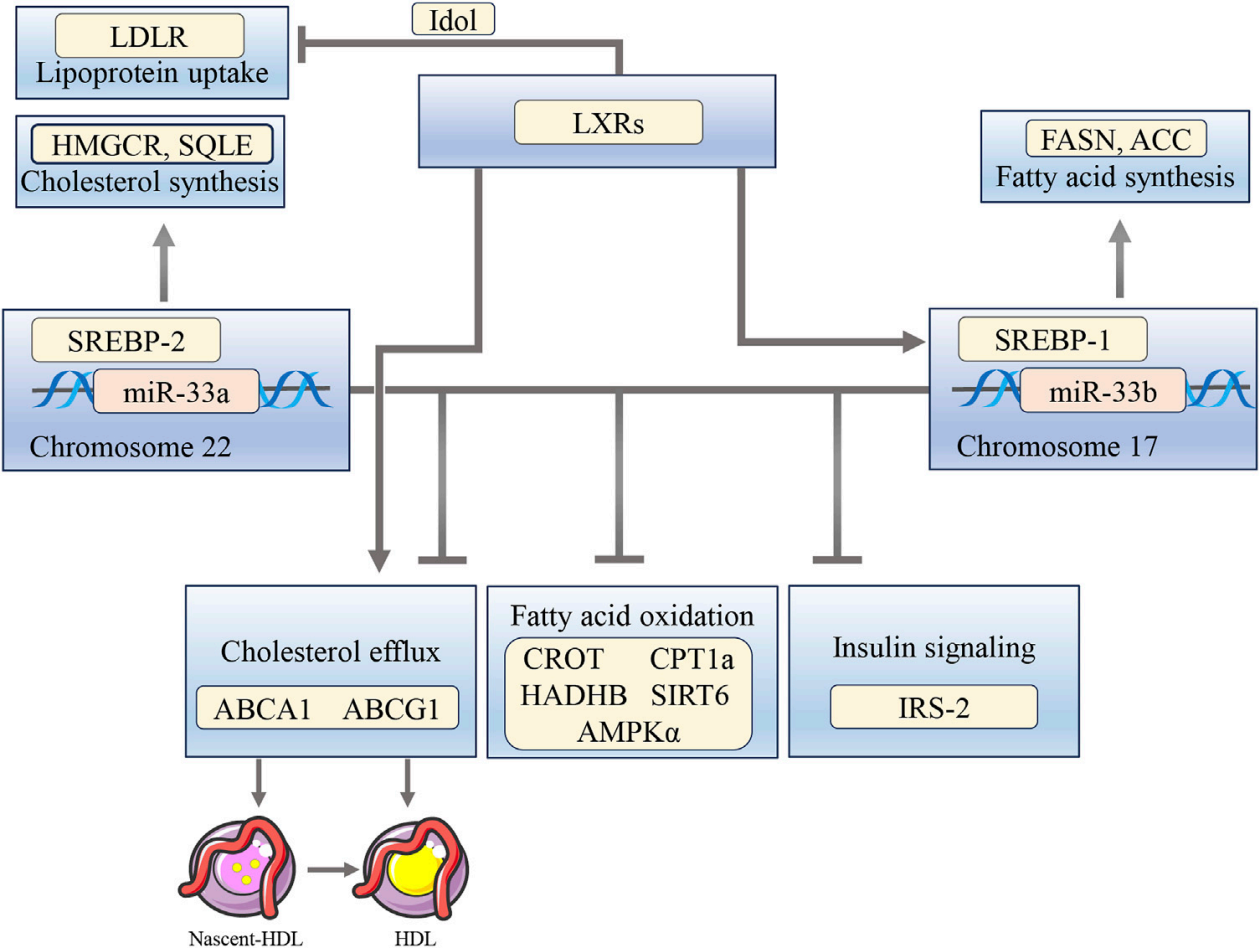
The interplay between miR-33, SREBPs, and LXRs influences the regulation of cholesterol, fatty acid oxidation, and insulin signaling pathways. miR-33a and -b target ABCA1 and ABCG1 to inhibit cholesterol efflux, as well as CROT, CPT1a, HADHB, SIRT6, and AMPKα to suppress fatty acid oxidation. They also target IRS-2, leading to reduced insulin signaling pathways. Activation of LXRs stimulates ABCA1 and ABCG1, induces SREBP-1c expression, and inhibits LDLR through Idol.

Humans have two forms of miR-33 genes: miR-33a and miR-33b. miR-33a is encoded in intron 16 of the SREBP-2 gene on chromosome 22, and miR-33b is encoded in intron 17 of the SREBP-1 gene on chromosome 17 (Dávalos et al., 2011). Similar to other intron miRNAs, miR-33 is often co-transcribed with its host gene SREBPs, and it targets genes related to the cholesterol output, ABCA1 and ABCG1 (Rayner et al., 2010). ABCA1 facilitates the transfer of cholesterol and phosphatidylcholine to lipid-free apolipoprotein A-I, leading to the formation of nascent HDL. On the other hand, ABCG1 is responsible for the transfer of cholesterol, phosphatidylcholine, and sphingomyelin to nascent HDL and HDL (Rosenson et al., 2016). Under conditions of sterol depletion, when SREBP-2 is activated to increase cholesterol biosynthesis and uptake, miR-33 inhibits the efflux of cholesterol from the cell by targeting ABCA1 and ABCG1. Conversely, when cellular cholesterol levels are high, SREBP-2 processing is inhibited, leading to reduced miR-33 expression. In this state, LXRs are active, leading to the activation of ABCA1 and ABCG1 by binding to LXR response elements in the gene promoter. This activation affects HDL levels and the removal of cholesterol (Price et al., 2021; Matsuo, 2022). In addition, LXR can hinder the LDLR pathway by transcriptionally stimulating the expression of Idol, which promotes the degradation of LDLR and hence reduces the uptake of LDL (Zelcer et al., 2009). miR-33a and -b also target key enzymes involved in the regulation of fatty acid oxidation, including carnitine O-octanoyltransferase (CROT), carnitine palmitoyltransferase 1A (CPT1a), hydroxyacyl-CoA-dehydrogenase (HADHB), Sirtuin 6 (SIRT6), and AMPKα. Additionally, miR-33a and -b also target the IRS-2, which is an important component of the hepatic insulin signaling pathway (Dávalos et al., 2011) (Figure 3).

Marquart et al. discovered that when C57BL/6J mice were injected with a miR-33 adenovirus vector (2 × 10^9 pfu), the levels of liver ABCA1 and serum HDL decreased. However, when the mice received a continuous tail vein injection of anti-miR-33 antisense oligonucleotide (ASO) (5 mg/kg/day) for 3 days, there was a significant increase in liver ABCA1 expression and serum HDL levels after 5 or 12 days of ASO infusion (Marquart et al., 2010). The results above suggest that miR-33 may control the balance of cholesterol and the metabolism of lipoproteins by suppressing the expression of ABCA1.

### 5.2 miR-122

miR-122 is ubiquitously expressed in the liver of vertebrates. It is estimated that miR-122 accounts for approximately 70% of all liver miRNAs and plays a variety of functions in liver physiology and pathology (Moore et al., 2011; Bandiera et al., 2015). miR-122 was the first identified miRNA involved in the regulation of total serum cholesterol and liver metabolism, and it is the most studied hepatic miRNA to date (Moore et al., 2011).

Pharmacological and genetic suppression of miR-122 is known to impair systemic and hepatic lipid metabolism, iron homeostasis, and hepatocyte differentiation (Thakral and Ghoshal, 2015). Esau et al. conducted an experiment where they fed C57BL/6J mice a diet heavy in fat for 19 weeks. Afterward, the mice were injected with 12.5 mg/kg of miR-122 ASO into their peritoneal cavity twice a week for five and a half weeks. After 2 weeks, this led to a substantial reduction in blood lipids and liver steatosis (Esau et al., 2006). Research has demonstrated that miR-122 controls the expression of luteinizing hormone receptors in rat ovaries via activating SREBP (Menon et al., 2015).

Additionally, miR-122 inhibition caused a significant decrease in the downstream pathways regulated by SREBP, such as HMGCS1, HMGCR, DHCR7, and SQLE (Rotllan and Fernández-Hernando, 2012). In mice with germline deletion of miR-122a, the downstream target genes FASN and SCD-1 of SREBP-1 were significantly reduced (Tsai et al., 2012). Although the regulation of lipid metabolism by miR-122 is complex, this microRNA does inhibit the downstream pathway of SREBP regulation. Further research is needed to determine the specific mechanism.

## 6 Discussion

With the rapid aging of the global population and the further implementation of clinical guidelines, polypharmacy is becoming more and more common, and a series of problems like adverse drug reactions and drug damage have become more apparent. At present, there is no effective method to solve polypharmacy dilemma in clinical practice. SREBPs and related pathways are critical for MS-related diseases. In summary, we have outlined the mechanism of SREBPs and their associated pathways, specifically in the context of prevalent metabolic disorders. Additionally, we have discussed the inhibitors commonly used to target these pathways and the regulatory miRNAs involved. The findings indicate that SREBPs and their associated pathways offer substantial potential as targets for the development of a single drug to treat a variety of diseases effectively. This provides a novel idea for solving the clinical polypharmacy dilemma. Although several studies have demonstrated the possibility of drug development targeting SREBP-related pathways in different ways *in vivo* and *in vitro*, further work is required.
